# When the orbital degree of freedom meets higher-order topology

**DOI:** 10.1038/s41377-023-01152-z

**Published:** 2023-04-28

**Authors:** Jiazheng Li, Meng Xiao

**Affiliations:** 1 Key Laboratory of Artificial Micro- and Nano-structures of Ministry of Education and School of Physics and Technology, Wuhan University, Wuhan, 430072 China; 2 Wuhan Institute of Quantum Technology, Wuhan, 430206 China

**Keywords:** Photonic devices, Nonlinear optics, Photonic crystals

## Abstract

The orbital degree of freedom (ODoF), which has a significant impact on exotic quantum states of matter and solid-state materials, has now been combined with higher-order topology. The experimental realization of a photonic p-orbital higher-order topological insulator can lead to exploring a wide range of novel topological phases involving the ODoF.

The orbital degree of freedom (ODoF) is a crucial attribute of solid-state materials and artificial structures such as photonic crystals and phononic crystals. The ODoF refers to the shape and orientation of the wave function in the crystal. Depending on its energy, a state may occupy the s-orbital (spherical), p-orbitals (dumbbell-shaped), or others. Several unconventional effects, including orbital hybridization and anisotropic coupling, need to be considered when the ODoF is introduced^1^. On the other hand, the recent attention of researchers in topological phases has been largely drawn to higher-order topological phases (HOTPs). HOTPs go beyond conventional bulk-boundary correspondence, and a nontrivial HOTP gives rise to topologically protected waves at higher co-dimensional boundaries, such as corners or hinges^2^. Therefore, HOTPs offer a new approach to confine waves in extremely small mode volumes in a topologically robust manner. This unique feature could enable many potential applications, including low-threshold lasers^3^ and high-quality factor nanocavities^4^. Now writing in eLight^5^, Zhigang Chen and Hrvoje Buljan groups explore the interplay between ODoF and HOTPs. They report the first experimental realization of p-orbital higher-order topological insulators (HOTIs) using photonic breathing Kagome lattices (BKLs) (see Fig. 1, left panel).

**Fig. 1:**
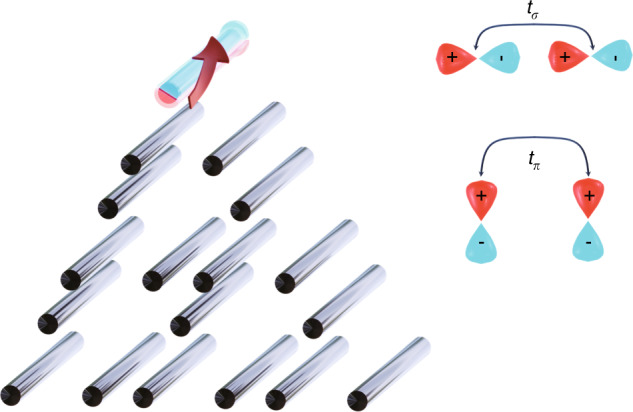
Left panel shows a p-orbital corner state in a nontrivial BKL. Under a certain nonlinearity, a tilted diploe-like corner mode rotates towards a prefer direction while propagating forward. Right panel illustrates anisotropic couplings induced by the orbital degree of freedom

The authors unveil the topological origin of orbital zero-energy corner modes by demonstrating that the p-orbital BKL Hamiltonian is topologically equivalent to a direct sum of two decoupled Hamiltonians. However, orbital hybridization induced by ODoF makes it impossible to associate each band with a certain subspace and uncover the topology behind bulk polarization. To reveal this system’s “hidden” topology, the authors proposed a generalized winding number in the presence of *C*_3_ rotational symmetry and the generalized chiral symmetry^6^. In addition, the robustness of orbital zero-energy corner modes under perturbations has been numerically analyzed. The authors show that besides *C*_3_ and the generalized chiral symmetry, an unconventional orbital-hopping symmetry that requires two orthogonal p-orbitals (see Fig. 1, right panel) to exhibit the same variation under lattice breathing is essential to enforce the topological protection of the p-orbital HOTI.

Using the continuous-wave laser writing technique, the authors fabricate nontrivial and trivial photonic BKLs for experimental control investigation. Writing waveguides in a nonlinear photonic crystal gives the researchers a nice platform to investigate the interplay of ODoF, HOTPs, and nonlinearity. Probe beams modulated into dipole-like distributions are utilized to selectively excite p-orbital corner states, and an external static bias electric field is employed to control the nonlinear coefficient together with the beam intensity^7^. Besides selectively exciting either of the two orbital corner modes, the authors have also observed a nonlinearity-induced dynamical rotation of orbital corner modes, where an oblique input beam rotates towards the favored orientation (see Fig. 1, left panel).

Undeniably, the interplay between ODoF, HOTPs, and nonlinearity sheds light on exploring novel features in photonic structures that may further facilitate applications in integrated photonics. The present work plays a critical role in ushering us into this area, with many exciting phenomena awaiting discovery. For example, solitons, a hallmark of nonlinear systems, may have an exciting interplay with orbital corner modes.
